# Characterising a novel mouse model with a mutated ciliopathy gene (*Cep290*) leading to Joubert Syndrome

**DOI:** 10.1186/2046-2530-1-S1-P89

**Published:** 2012-11-16

**Authors:** AM Hynes, CA Miller, L Eley, RJ Simms, K White, C Miles, JA Sayer

**Affiliations:** 1 Institute of Genetic Medicine,Newcastle University, International Centre for Life, UK; 2 Electron Microscopy Core for PKD Research, Department of Anatomy & Cell Biology, Indianapolis University School of Medicine, USA; 3 Electron Microscopy Research Services, Catherine Cookson Building, Newcastle University Medical School, UK

## 

Joubert syndrome (JBTS) is an inherited ciliopathy leading to a cerebellum-retinal-renal syndrome. Recent genetic advances have allowed positional cloning and identification of numerous JBTS genes. *CEP290*, one of the JBTS genes identified, (alias *NPHP6*) encodes a centrosomal protein and accounts for 7% of patients with Joubert syndrome. We have identified a murine Embryonic Stem (ES) cell line containing a *Cep290* “gene trap” using data base searches. ES cells were cultured before injecting into murine blastocysts to create chimaeric mice. Chimeras were bred to produce viable, healthy heterozygous mutant mice. Heterozygous mutant mice have been intercrossed to produce mice homozygous for the *Cep290* truncating mutation. *Cep290-/-* animals (homozygous for the gene trap *Cep290*) exhibit a cortico-medullary cystic kidney disease commencing from birth. Histological examination reveals that these cysts are collecting duct in origin, staining positively for aquaporin-2 and -3. In this study the cilia were investigated in cystic *Cep290-/-* animals using Electron Microscopy (EM) analysis. Scanning electron microscopy (SEM) identified that cilia were evident within renal tubules in *Cep290-/-* animals. Once cilia were identified in *Cep290-/-* animals Transmission Electron Microscopy (TEM) was carried out to investigate cross sections of the collecting duct cilium in cystic and non-cystic kidneys. TEM analysis identified tubular basement membrane disruptions in *Cep290-/-* animals. The *Cep290-/-* mouse described provides an excellent model to investigate the mechanisms involved in cyst formation and to test novel therapeutic agents.

